# System-wide modeling approaches for integrating supply chains into food security analysis: lessons from Uganda 2016–2020

**DOI:** 10.1057/s41271-026-00644-7

**Published:** 2026-08-12

**Authors:** Luc Gendre, Courtney Blair, Tristan Downing, Jarrod Goentzel, Erica Gralla, Timothy Russell

**Affiliations:** 1 Massachusetts Institute of Technology, Cambridge, MA United States; 2 George Washington University, Washington, DC, United States

**Keywords:** Food security, Supply chain, System dynamics, Systems thinking, Causal loop diagrams

## Abstract

Supply chains are central to food security modeling, particularly in famine-prone settings, yet traditional analyses rarely adopt a system-wide perspective that accounts for their influence. One reason is that conventional predictive approaches used in supply chain analysis and systems modeling are difficult to calibrate and validate in contexts marked by sparse data and rapidly changing conditions. Drawing on a 6-year collaboration with USAID in Uganda, we argue that in such settings, an adapted system mapping approach can enable system-level analysis and produce actionable insights. We present our approach and examine its application to the Karamoja region of Uganda, where USAID sought to strengthen household resilience. We demonstrate the value of descriptive and prescriptive models in contexts where prediction is not feasible. In Karamoja, this approach highlighted the central role of supply chains in resilience outcomes and supported collective sense-making and collaboration among practitioners.

## Key messages

- Supply chains are both foundational to food security and a practical entry point for broader system modeling. Because they already endogenize many of the actors, flows, and constraints shaping food systems, they offer a concrete anchor for system-wide analysis.
- System modeling remains valuable in humanitarian settings when the tools are adapted to the context. Even where data are too limited for calibration and forecasting, tailored modeling approaches can still clarify problems, reveal interdependencies, and identify plausible intervention points.
- The value of modeling often extends beyond prediction as the process itself can strengthen coordination. Collaborative system mapping can help practitioners develop a shared understanding of the system, surface gaps and complementarities across interventions, and support more coherent collective action.

## Introduction

Supply chains function as key enablers of food systems by facilitating the flow of goods and services from producers to consumers, thereby playing a vital role in food security. When disease, disaster, or conflict disrupts supply chain operations, shortages of essential products can escalate into severe hunger crises. A contemporary example is the war between Russia and Ukraine, which disrupted exports of maize, wheat, and sunflower oil, cutting off critical supply routes to the Middle East and Africa and leading to food scarcity [1].

Conversely, such crisis impacts can be mitigated by either strengthening supply chain resilience or creating parallel, “replacement” systems (humanitarian supply chains) to effectively deliver food and essential supplies to vulnerable populations. Separate streams of the supply chain literature have addressed supply chain resilience, with a primary focus on commercial actors (e.g., [2]); as well as humanitarian logistics, which focuses on designing, managing, and adapting intervention supply chains to ensure effective delivery during crises [3, 4].

Yet, a system-wide perspective, essential to fully capture the relationships between supply chains and food security, is often lacking in traditional analyses [5–7]. One reason may be contextual: predictive modeling tools are difficult to rigorously calibrate and validate in the conditions that characterize famine-prone settings. In such contexts, data are often sparse, noisy, rapidly outdated, and uneven across locations. These features make fully quantified, traditionally parameterized system dynamics and related simulation models difficult to validate scientifically and therefore reduce their operational value.

We argue that in data-scarce humanitarian settings, the value of system modeling may lie more in the descriptive and prescriptive capabilities enabled by the shared process of constructing, iterating, and engaging models to support sense-making, exploration of system mechanisms, and cooperative intervention design. The system maps created using our approach are fundamentally descriptive: defining the problem, identifying relevant variables, and mapping their relationships. According to Mitroff et al. [8], such conceptual models are crucial for avoiding “errors of the third kind”: addressing the wrong problem entirely. Where sufficient data exist, these models may also provide the basis for predictive models that can be calibrated and validated more formally. Yet prescription does not depend on prediction alone: even in the absence of sufficient validation data, descriptive conceptual models can still support action by helping actors identify intervention points, reason through system mechanisms, and design more coherent responses [9].

We begin by presenting a research partnership with the United States Agency for International Development (USAID) in Uganda, which provided rich context for developing a novel approach derived from traditional systems engineering methods and tailored to data-scarce environments. Next, we illustrate how models were used to illustrate the centrality of supply chains to food security and how the modeling process itself supported shared understanding and coordination among actors. Through this case study, we demonstrate how researchers and policymakers can integrate supply chain modeling into a systems approach to famine, while highlighting the value of cross-disciplinary collaboration and emphasizing the importance of adapting traditional models to humanitarian contexts.

## Background

This paper draws on a 6-year collaboration among a research team from the Massachusetts Institute of Technology (MIT), The George Washington University (GW), and the USAID Mission in Uganda [10]. The goal of the collaboration was to apply supply chain modeling and systems engineering tools to complex local systems in Uganda, to better understand, monitor, and predict how these systems would evolve and change.

Our team developed new approaches, grounded in existing systems methods, to support USAID to plan, assess, and adapt the activities under its Feed the Future Value Chain project, which focused on strengthening the agricultural market system in Uganda. One such approach was a novel system mapping technique that was designed to be accessible to development practitioners and could be applied in data-poor environments, termed the “System Pathways Toolkit.” [11–13].

We built and refined this approach through the process of creating a detailed map of Uganda’s agricultural market system, which is documented in [14–18]. The physical supply chains that undergird food production formed the backbone of this map: the movement of agricultural inputs from manufacture to distribution to cultivation, and the movement of outputs from field to aggregator to consumer. This organizing principle improved the accessibility of the map, as it reflected tangible processes that practitioners were familiar with.

Through this foundational work on the agricultural market system, we were able to demonstrate both that supply chains are essential to market development and food security, and that system mapping is an invaluable tool to visualize, communicate, and monitor this relationship. This broader relevance prompted USAID to extend its use of system mapping beyond its original market development application. In 2019, it invited us to apply it to its work in Karamoja, Uganda, to examine household resilience in a region vulnerable to humanitarian crises, including famine.

The Karamoja region is characterized by traditional pastoralist livelihoods, and lags behind the rest of the country in poverty reduction and economic development. It is a semi-arid region that is drought-prone and highly vulnerable to climate change, with a history of armed violence [19]. The region experiences recurring famine and acute food insecurity and has a long history of humanitarian support [20]. It is undergoing a gradual transition to agro-pastoralist and agriculture-focused livelihoods, and USAID and other donors have been implementing a mix of humanitarian and development programming to support this transition [21].

Humanitarian and development assistance efforts have historically operated in silos [22], though efforts have been made over the past decade to improve coordination at the “humanitarian-development-peace nexus” [22, 23]. USAID/Uganda was determined to improve collaboration across its Global Food Security Strategy (GFSS) portfolio in Karamoja, which included USAID staff and implementing partners working on humanitarian (Food for Peace) and development (Feed the Future) programming. USAID adapted the cluster approach used in humanitarian contexts to establish the Karamoja Resilience Cluster, which focused on improving the resilience of Karimojong communities in three districts in the region.

Our objective was twofold: first, to generate insights for USAID using a systems approach to analyze Karamoja through a supply chain lens; and second, to support greater collaboration in the Karamoja Resilience Cluster.

## Methods

To design interventions and monitor their implementation, development professionals have traditionally relied on “logframes” and results chains to depict the causal links between interventions and outcomes. These tools are often too linear and restrictive to independently encompass the diverse factors influencing system behavior. However, they align well with the system dynamics modeling methods that Besiou and Van Wassenhove [24] position as central to decision-making in humanitarian operations, by simulating how different components within a complex system interact and evolve over time. Such an approach provides a framework to analyze the long-term behavior of complex systems and to develop strategies to influence outcomes in dynamic environments [25].

**Fig. 1:**
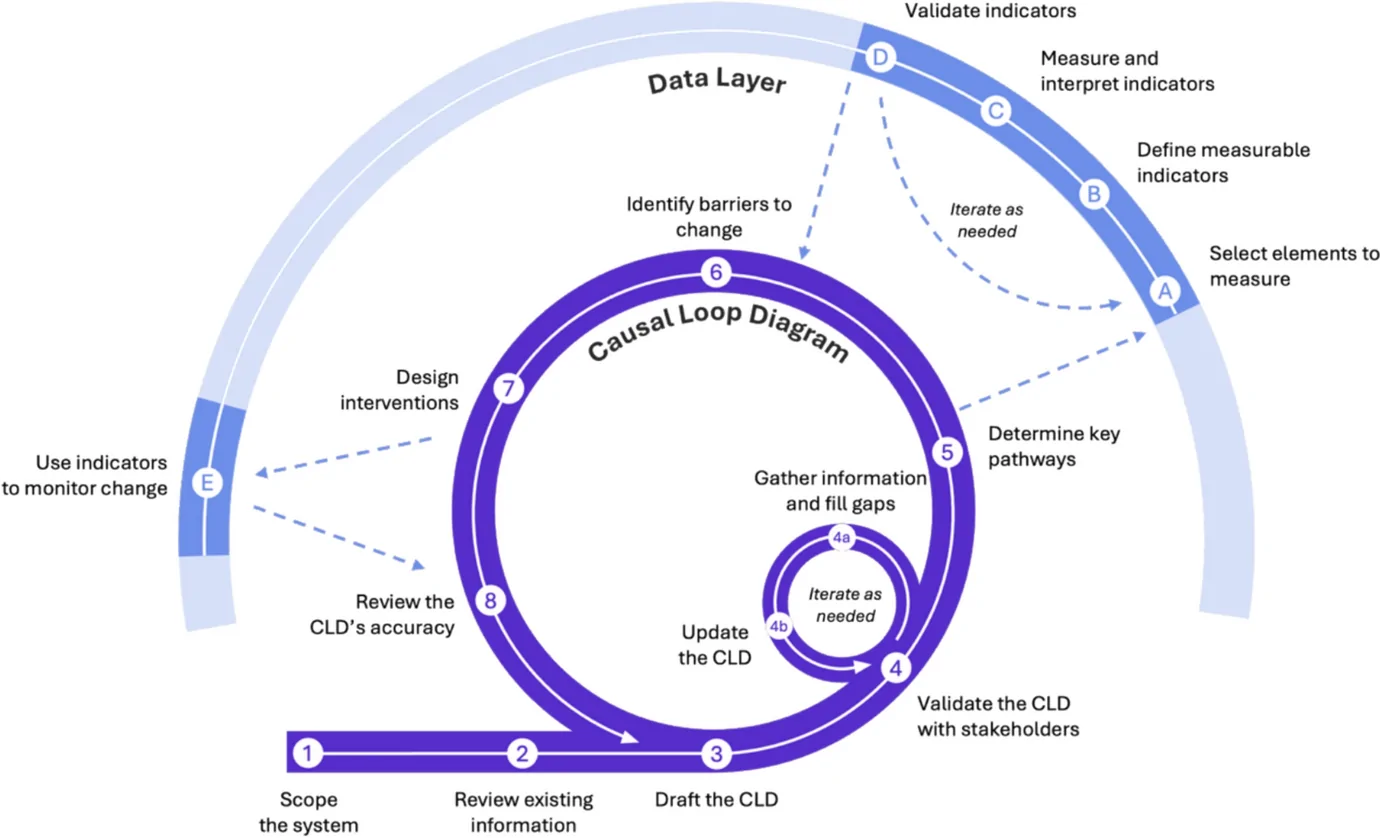
CLD development and measurement process

We developed the System Pathways Toolkit to bridge the gap between linear results chains and traditional system dynamics. It is a diagramming method that adapts the causal loop diagram (CLD), a recognized systems method that visually maps important concepts and their causal relationships [25]. By representing and connecting variables including behaviors, relationships, and conditions that can influence the system, the maps reveal feedback loops as well as barriers and pathways to change. Our approach also allows for monitoring and adaptation through the addition of a data layer, which links the available data to customized indicators. More detail is provided in Blair et al. [26] and in our Toolkit [11]. We follow a collaborative, iterative process to develop these maps, as described in Fig. 1; for this engagement, we executed steps one through six.

The first system map we developed for the Karamoja context represented the building blocks of household resilience. In this setting, a resilient household was defined as one that “maintains its well-being in the face of shocks and stresses.” The Karamoja Household Resilience System Map grew out of a workshop hosted in June 2019 for Uganda’s GFSS portfolio, which identified a need to better understand the factors underpinning resilience, particularly the interaction between humanitarian support, livelihoods, and market access [27]. To develop the map, our team worked with eight USAID implementing partners in the Karamoja Resilience Cluster, together with stakeholders from other donor-funded activities. An initial version was produced following a first meeting in which we facilitated discussions to elicit practitioners’ views on the main drivers of household resilience in Karamoja. All maps were developed on the Kumu platform, which we selected because it is free, accessible, and relatively easy for non-specialists to use. The map was then iterated and refined: Cluster members were invited to expand and edit it themselves, while our team reviewed partners’ theories of change, the literature on household resilience, and prior research on Karamoja. In particular, the work of Maxwell and Wiebe on the livelihoods cycle informed the structure of the map [13]. We also held intensive consultations, including small-group mapping sessions, both to build participants’ familiarity with system mapping and to verify that the map accurately reflected their perspectives. In the final round of meetings before the workshop, the map was validated and partners’ specific interventions were placed onto it. The full process is documented in a published case study [28]. The final map is available online [29], together with supporting guidance on how to interpret it [30, 31].

**Fig. 2:**
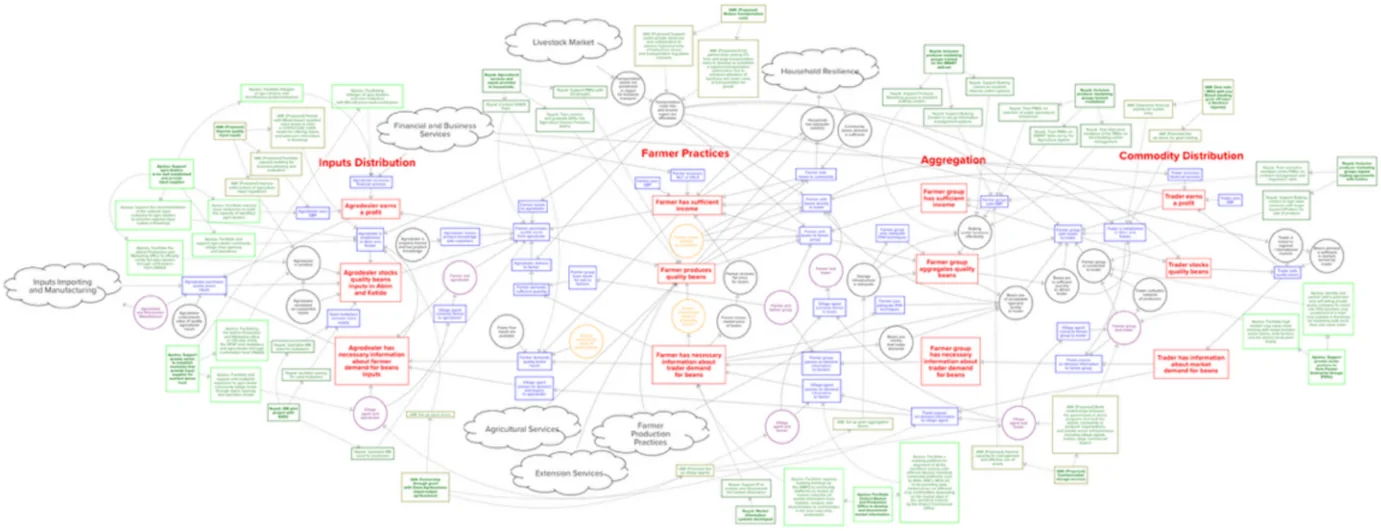
Karamoja beans market system map

**Fig. 3:**
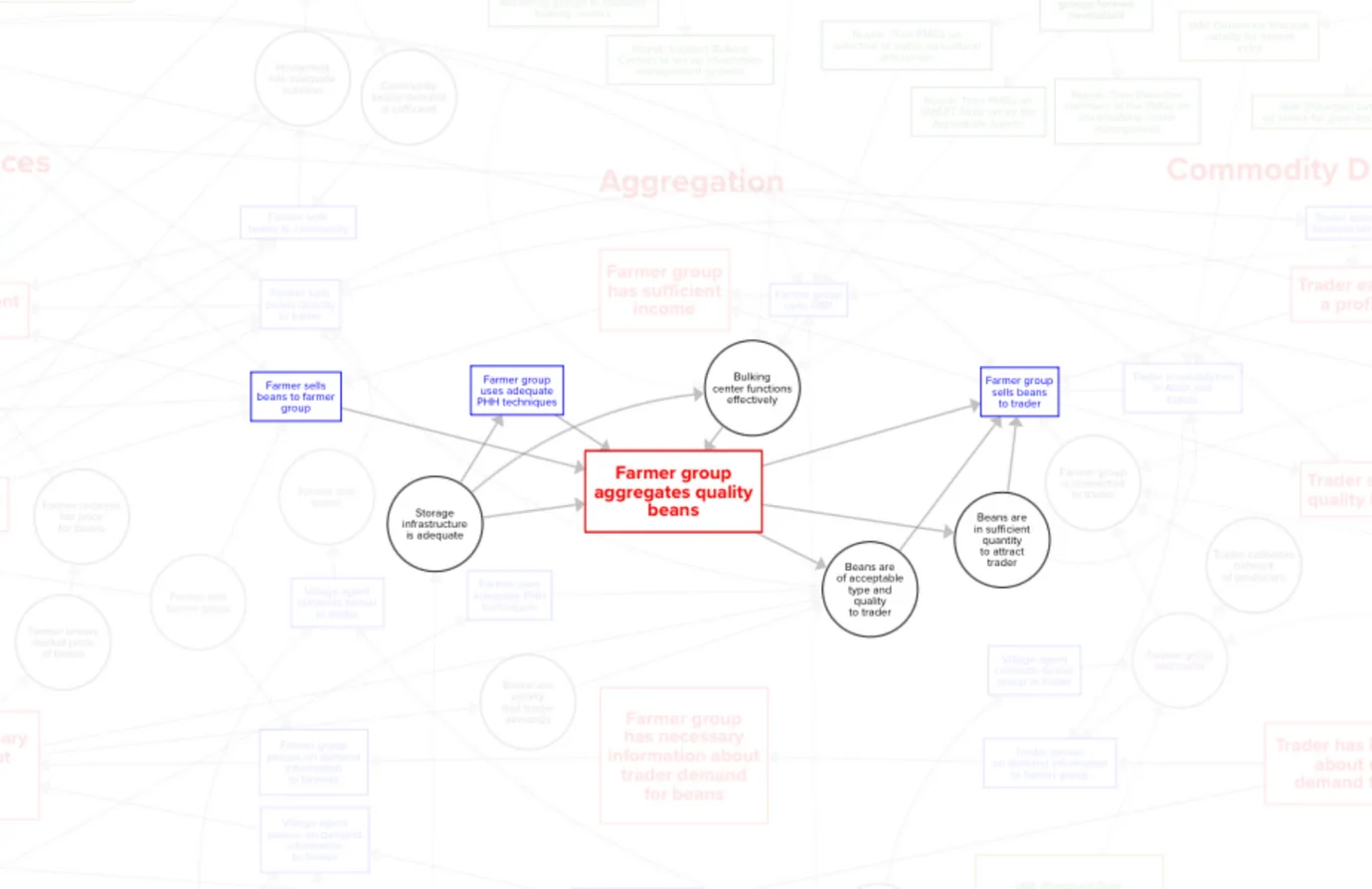
Close-up of one section of the Karamoja beans market system map

Following the positive reception of the initial map, our team was asked in 2020 to apply the same methodology to specific market systems in Karamoja, leading to two further maps developed with the Cluster: the Karamoja Livestock System Map [32], which focuses on pastoralist livelihoods in the region, and the Karamoja Beans Market System Map [33], which focuses on the production of iron-rich beans in two districts (Figs. 2 and 3). The purpose of the livestock system map was to serve as a communication tool for explaining the dynamics of pastoralist livelihoods in the region, while the iron-rich beans map was created to support collaboration between interventions focused on encouraging production of this biofortified crop. Both also demonstrated how these localized markets were linked to broader national supply chains: in the livestock case, for veterinary supplies and live animals; for iron-rich beans, access to inputs and produce markets.

**Fig. 4:**
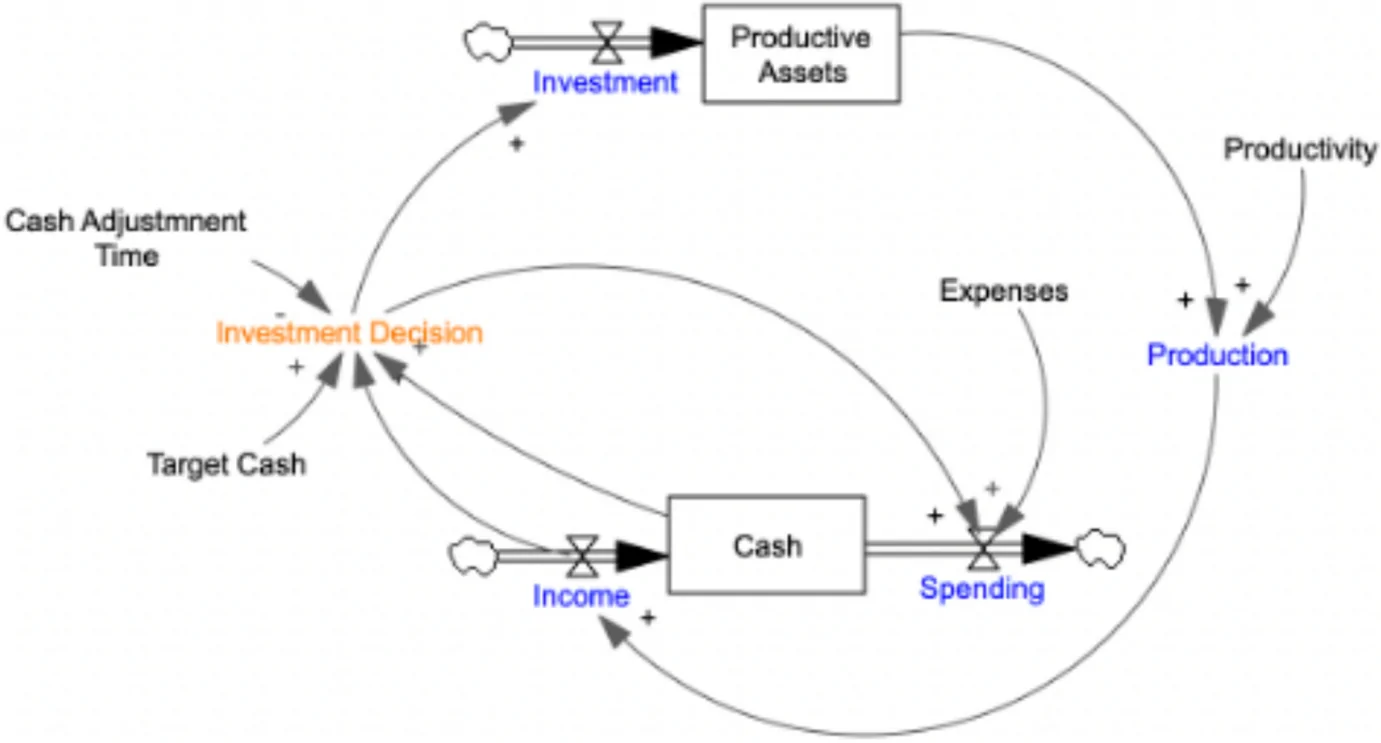
Example of system dynamics modeling in Karamoja: income increases cash reserves, enabling investment in productive assets, which boosts production and productivity; this in turn generates more income, while spending and expenses draw down cash, creating a balancing loop that adjusts investment levels to maintain financial stability over time

As a final extension of this work, we also developed a simple system dynamics simulation model of Maxwell and Wiebe’s livelihoods cycle for household resilience [34]. System dynamics simulation modeling is a well-established methodology [25] that uses mathematical simulations of an interconnected system to predict its behavior over time. These simulations often build on causal loop diagrams and are applied to systems with complex feedback dynamics when sufficient data is available. In this model, illustrated in Fig. 4, a household is resilient if it can recover and continue growing its assets in the face of a shock or stress. The model was notional, as data was insufficient to properly calibrate and validate it. Indeed, even the broader concept of sustainable livelihoods has had little numerical modeling attention, with previous work typically focusing on narrow parts of the concept [35]. Yet, assembling a scientific model for the livelihoods cycle would enable a better understanding of household adaptation, which is key to anticipating the intended and unintended consequences of interventions designed to increase household resilience in the face of food insecurity [31].

## Results

This section is organized around two main findings. First, supply chains proved to be both a useful anchor for wider system modeling and a recurring source of insight into the drivers of food security outcomes. Second, the process of building and using these maps enabled collective sense-making and more coordinated action among stakeholders.

### Part 1: from supply chains to the wider system, and back again

Throughout our engagement in Karamoja, supply chains served not only as a useful entry point for mapping the wider system, but also frequently emerged as a central finding of the analysis, as they were often identified as key constraints on, or enablers of, food security and resilience outcomes. The mapping highlighted flows of agricultural inputs, crops, livestock, food, pharmaceuticals, and other goods and services into and out of the region as critical both to crisis response and to longer-term resilience.

**Fig. 5:**
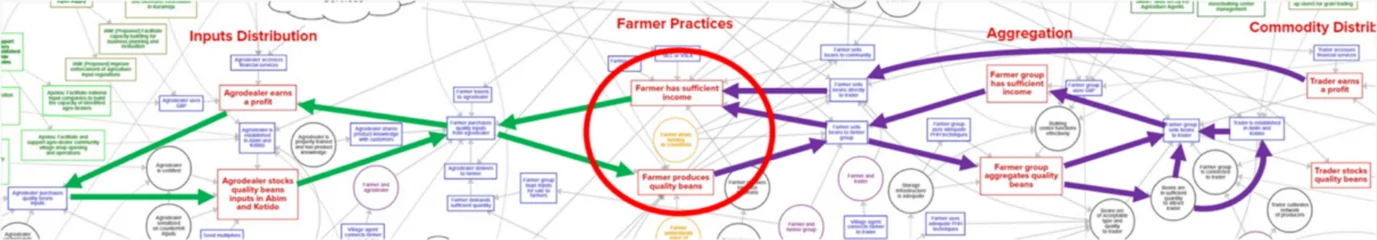
Feedback loops in the supply chain for iron-rich beans

Building outward from individual supply chains to the wider system also made it possible to uncover dynamics shaping household resilience. The Karamoja Beans Market System Map enabled the practitioners to identify interconnected causal loops within the markets (see Fig. 5). For instance, in the supply chain for iron-rich beans, successful aggregation by farmer groups is more likely to attract traders, which in turn encourages more farmers to participate in future seasons. However, without traders, farmers hesitate to engage in collective marketing, and without supply, traders are reluctant to enter the district. The success of this loop depends on the alignment of both elements, but also on the reliable production of iron-rich beans, which requires an adequate supply of inputs, forming another feedback loop between farmers and input dealers. By using these models, USAID partners learned that facilitating positive causal loops is crucial for developing robust supply chains that enhance household resilience.

**Fig. 6:**
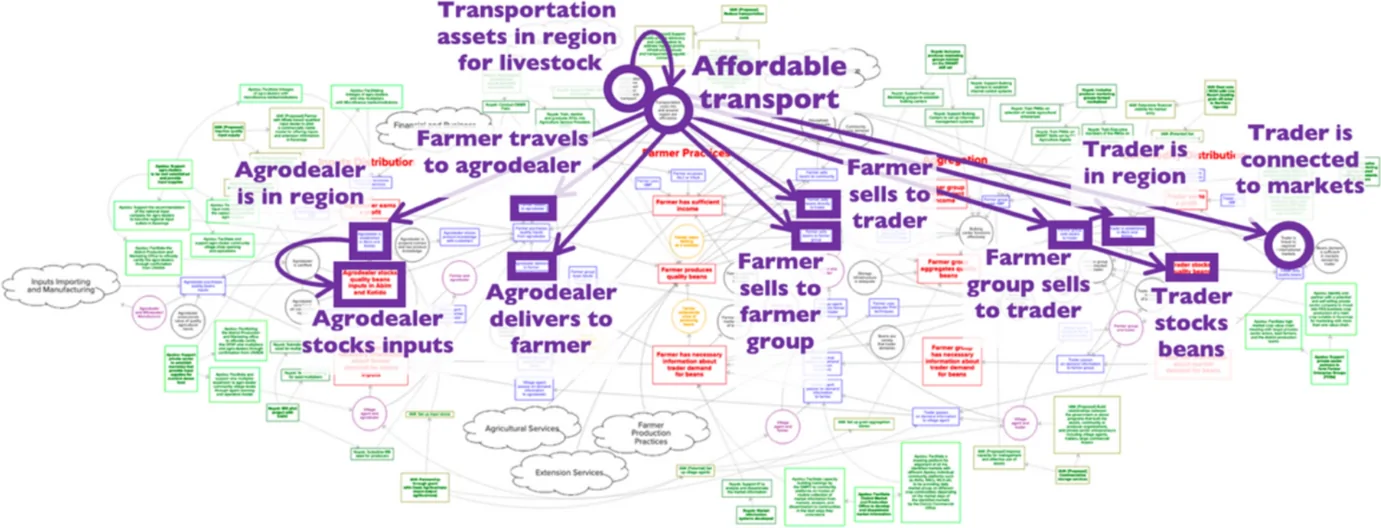
Transportation effects highlighted on the Karamoja Beans Market System Map

Modeling the wider system also made it possible to identify synergies across supply chains that would have remained invisible in a siloed value chain analysis. For example, the map showed how the supply chain for iron-rich beans could build on existing actors, infrastructure, and market linkages in more established chains such as sorghum, maize, or sunflower. This included opportunities to piggyback on trader networks, share transportation assets, exploit economies of scope in freight, and align with interventions led by other donors. Extending the analysis beyond an individual supply chain also showed that many of these supply chains depended on common enabling functions, especially transportation, which appeared repeatedly across the conceptual model (Fig. 6). This made clear that transport operated as a system-wide constraint affecting actors both upstream and downstream of the farmers. These effects collectively slowed market development by impeding not only the flow of inputs and outputs, but also the flow of money and market information. By making these interdependencies visible, the map helped identify opportunities for complementary interventions, ranging from transport infrastructure improvements to measures that improve freight market performance and truck utilization across commodities.

### Part 2: enabling collective sense-making and improved collaboration

System models can support action in a direct and relatively conventional way. For instance, our simple system dynamics model of household resilience later informed a broader model for the International Committee of the Red Cross (ICRC) in Northeast Nigeria, integrating household needs and supply chain dynamics to explore the appropriate mix of humanitarian response options, including in-kind assistance, cash assistance, and credit for supply chain actors [36].

But for our Karamoja maps, the contribution lay not only in their prescriptive value, but also in the shared process of building and iterating them. That process created a shared understanding of resilience among a variety of actors and enabled them to reason collectively about how their interventions interacted across the wider system. In these respects, the work directly enabled what USAID referred to as “Collaboration, Learning and Adaptation,” an approach to improving development outcomes through adaptive management. Table 1 summarizes the main ways in which the mapping work proved useful. We elaborate below on several examples of how these contributions materialized through our engagement with the Karamoja Cluster.

**Table 1:** Potential contributions of the mapping work to collaboration, learning, and adaptation

Category	Mapping-enabled action
Collaboration	Build common understanding through participatory mapping
	Identify opportunities for collaboration and complementarity
	Communicate theories of change with other stakeholders
Learning	Learn how a system is structured and functions
	Identify gaps in understanding of the system or in available data
	Develop a learning agenda based on gaps identified
	Monitor system change over time
Adaptation	Test theories of change
	Identify barriers to system change or drivers of unexpected results
	Rapidly evaluate the impact of a shock

In January 2020, we hosted the first workshop of the Karamoja Resilience Cluster with support from the Uganda Learning Activity. The workshop gave participants an opportunity to better understand one another’s work and to begin defining how the Cluster would operate going forward. The Karamoja Household Resilience System Map served as a key organizing tool, structuring communication and framing discussion. Additional detail on the workshop outputs is available in [37, 38].

The mapping process generated a number of positive outcomes at the workshop, which were not quantified directly but captured from post-event feedback. First, stakeholder participation in the creation of the map helped promote a collective understanding of the system and of the drivers of resilience. The map showed how the activities’ results chains were linked to the high-level outcomes that they cared about, which helped them to better understand the system, by locating their own work within it and framing their contribution through their existing understanding. This enabled the stakeholders to better understand each other’s mindset, perspective, and dynamic hypotheses about system change. As one participant noted, it was useful to “see how moving parts work together”.

The workshop also gave implementing partners their first structured opportunity to learn about one another’s work. Because the map captured all USAID-funded activities in the Cluster districts, participants could quickly identify where interventions were concentrated, who was working on related issues, and where opportunities existed for synergy, collaboration, or sharing of resources and expertise. We also encouraged participants to think in terms of “complementarity,” that is, ways in which interventions might support one another indirectly by acting on different parts of the system that jointly shape a desired outcome. The workshop produced a documented set of specific collaboration opportunities, and a participant reported that they had “learned of other activities’ interventions and potential areas of collaboration”.

Beyond identifying specific opportunities for collaboration, the workshop also surfaced broader priorities for collective action by highlighting key barriers to change and gaps in the existing approach. In particular, participants identified water access, conflict, and gender issues as cross-cutting factors affecting multiple dimensions of household resilience. These insights were made more visible by the map, which encouraged participants to view resilience from a systems perspective rather than through isolated interventions. In this way, the map helped clarify programming needs and opportunities in the region, particularly for newer activities.

The Karamoja maps were intended to serve as the foundation for a further stage of work: layering available indicators onto the conceptual models to show how the system was evolving over time. In Blair et al. [26], we argued that this combination of structure and partial data can substantially improve the usefulness of causal maps in development and humanitarian settings, where information is often incomplete but still sufficient to highlight important patterns of change. That next step was not completed in Karamoja because the onset of COVID-19 led USAID to redirect attention toward the immediate pandemic response. As a result, our engagement with the Karamoja partners was cut short, and we could not follow the longer-term use of the maps or document their influence on programming.

At the same time, this shift provided an important demonstration of the broader value of the approach. Rather than continuing in Karamoja, we pivoted to the larger Uganda market system mapping work, which USAID used to support rapid assessment of COVID-related disruptions. This underscored one of the main arguments of this paper: when interventions must be adapted in response to a shock, it is highly valuable to begin from a shared understanding of how the system works. A common mental model makes it easier to assess which connections are likely to be disrupted, which actors or functions are most vulnerable, and where adaptation may be most effective. Thus, while this paper focuses on the contribution of adapted systems modeling to shared understanding and intervention design under relatively stable conditions, the same modeling logic also informed later crisis analysis. We leave to a separate paper the explanation of how rapid model adaptation early during the COVID-19 shock helped assemble unstructured data, define sentinel indicators, and anticipate emerging risks across the agricultural market system.

## Conclusions

Drawing on a multi-year collaboration with USAID/Uganda, this paper illustrates how efforts to understand and influence the dynamics of famine-prone systems can benefit from engaging cross-disciplinary system actors using tools that are tailored to humanitarian contexts. It also highlights the value of supply chains as an entry point for system modeling, as supply chains already encompass many of the actors, flows, and constraints that structure food systems, and their concrete, tangible nature makes them a practical foundation. We demonstrated how our approach enabled us to facilitate conversations and collaboration across the humanitarian-development-peace nexus, and drive deeper understanding of the complex dynamics that enable household resilience in areas with limited market opportunities.

A key limitation in such system modeling efforts is the reality of market fragmentation and data scarcity, which are often prevalent in regions that are vulnerable to famine. To overcome these challenges, we adapted modeling approaches [37] and guided policymakers in understanding their results to responsibly shape USAID activities in Uganda. In similar contexts, researchers should consistently characterize such data limitations and highlight modeling adaptations when providing directional guidance to policymakers.

The central lesson of this work, however, is that in such settings the value of system modeling may lie less in precise prediction than in the process of constructing, iterating, and using models to support inquiry and action. In our case, the maps helped actors develop a shared representation of the system, surface interdependencies that would otherwise have remained implicit, and identify opportunities for more coherent intervention. The policy implication is therefore not simply that organizations should possess a finished system map, but that they should engage in the collaborative process of building and improving models with descriptive and prescriptive value to enable collective sense-making, coordinated intervention, and system monitoring and adaptation.

## Data Availability

No datasets were generated or analysed during the current study.

## Abbreviations

CLA: Collaboration, learning and adaptation
CLD: Causal loop diagram
COVID-19: Coronavirus disease 2019
GFSS: Global food security strategy
ICRC: International Committee of the Red Cross
USAID: United States Agency for International Development
